# Pathway-based analysis of a genome-wide case-control association study of rheumatoid arthritis

**DOI:** 10.1186/1753-6561-3-s7-s128

**Published:** 2009-12-15

**Authors:** Joseph Beyene, Pingzhao Hu, Jemila S Hamid, Elena Parkhomenko, Andrew D Paterson, David Tritchler

**Affiliations:** 1 Biostatistics Methodology Unit, Research Institute, Hospital for Sick Children, 555 University Avenue, Toronto, Ontario M5G 1X8, Canada; 2 Dalla Lana School of Public Health, University of Toronto, Health Sciences Building, 155 College Street, Toronto, Ontario M5T 3M7, Canada; 3 The Centre for Applied Genomics, The Hospital for Sick Children Research Institute, 101 College Street, Toronto, Ontario M5G 1L7, Canada; 4 Division of Epidemiology and Statistics, Ontario Cancer Institute, 610 University Avenue, Toronto, Ontario M5G 2M9, Canada; 5 Department of Biostatistics, State University of New York at Buffalo, 249 Farber Hall, 3435 Main Street, Building 26, Buffalo, New York 14214-3000, USA

## Abstract

Evaluation of the association between single-nucleotide polymorphisms (SNPs) and disease outcomes is widely used to identify genetic risk factors for complex diseases. Although this analysis paradigm has made significant progress in many genetic studies, many challenges remain, such as the requirement of a large sample size to achieve adequate power. Here we use rheumatoid arthritis (RA) as an example and explore a new analysis strategy: pathway-based analysis to search for related genes and SNPs contributing to the disease.

We first propose the application of measure of explained variation to quantify the predictive ability of a given SNP. We then use gene set enrichment analysis to evaluate enrichment of specific pathways, where pathways, are considered enriched if they consist of genes that are associated with the phenotype of interest above and beyond is expected by chance. The results are also compared with score tests for association analysis by adjusting for population stratification.

Our study identified some significantly enriched pathways, such as "cell adhesion molecules," which are known to play a key role in RA. Our results showed that pathway-based analysis may identify other biologically interesting loci (e.g., rs1018361) related to RA: the gene (*CTLA4*) closest to this marker has previously been shown to be associated with RA and the gene is in the significant pathways we identified, even though the marker has not reached genome-wide significance in univariate single-marker analysis.

## Background

Rheumatoid arthritis (RA) is the most common systemic autoimmune disease characterized by chronic, destructive, and debilitating inflammation of joints and extra-articular tissues [1]. The disease affects 1% of the adult population worldwide and is significantly more prevalent in women (3 to 1 ratio) than men. It is believed that the contribution of individual genes to complex diseases such as RA, is generally modest and difficult to detect due to inadequate sample sizes compared with the large number of variables being tested for association. Currently, the literature on genome-wide association studies is focused on testing single-nucleotide polymorphisms (SNPs) for association using standard test statistic (for example, chi-square test statistic). The difficulty in detecting genes of modest effects may be one potential explanation for inconsistent results often seen in genetic association studies [2]. In a recent genome-wide study of RA with a relatively large sample size, Plenge et al. [3] identified a genome-wide significant association signal on chromosome 9 close to *TRAF1 *and *C5 *in addition to confirming known genes related to RA (e.g., *HLA-DRB1 *in major histocompatibility complex (MHC) region and *PTPN22*) [3].

In this paper, we propose a pathway-based approach to study sets of genes. The motivation for this is the belief that the mechanism of a complex disease such as RA may not be described fully by looking at gene-by-gene comparisons alone. Gene set analysis has been widely used in studies involving gene expression data; however, there are only a few such studies in a genome-wide association setting. We also propose two measures of explained variation that are appropriate for binary outcomes and summarize these measurements over all SNPs in and around a particular gene.

## Materials and methods

### Data description

The provided data is a subset of the Stage 1 genome-wide association study of RA previously analyzed by Plenge et al. [3]. After removing duplicated and contaminated samples, there were 868 cases from the North American Rheumatoid Arthritis Consortium (NARAC) and 1194 controls on which 545,080 SNPs had been genotyped.

### Quality control

The SNPs and samples fulfilling the following quality control requirements were included in our analysis: 1) call rate: SNP and sample call rates > 95%; 2) Hardy-Weinberg equilibrium: false-discovery rate (FDR) level in testing for Hardy-Weinberg equilibrium among controls > 0.2; 3) minor allele count > 10 copies, which is equivalent to a minor allele frequency of 0.24% (i.e, 10/(2 × 2062) = 0.24%). A total of 490,000 SNPs and 2062 samples met our quality control criteria and were used for further analysis.

### Adjustment for population stratification

It has been indicated that this data set is affected by population stratification, and this may lead to misleading association results if not taken into account. We followed an approach proposed by Price et al. [4] to adjust for population stratification. The steps involved can be summarized as follows: i) Using the SNP set remaining after applying our quality control criteria, we first removed SNPs in the MHC region (29-34 Mb on chromosome 6) and on the sex chromosomes. ii) For the remaining SNPs, we pruned them based on linkage disequilibrium using PLINK [5]. iii) This left around 220 k SNPs for which we calculated genome-wide pair-wise identity-by-state similarity matrix followed by multidimensional scaling analysis on the identity-by-state-based distance matrix. iv) We selected 15 significant principal coordinates (PCs) with *p*-value < 0.001. v) These 15 significant PCs were used in subsequent association analyses. It should be noted that these steps were only applied to select these 15 significant PCs. In the subsequent association analyses, we used all SNPs and samples that passed the quality control criteria.

### Univariate SNP association test

We performed score tests for association between RA status in patients and their genotypes, and adjusted for possible population stratification using the significant principal components. We used the '*egscore*' function in GenABEL R package [6], in which the population stratification method proposed by Price et al. [4] is implemented. We obtained the corresponding *p*-value and chi-square value for each SNP.

### Measures of explained variation

For a given SNP, let (*y*_*i*_, *x*_*i*_), *i *= 1, ..., *n *denote its *n *samples/observations, where *y*_*i *_denotes the outcome of the observation *i *(in this study, *y*_*i *_= 1 if the subject is a case and 0 otherwise) and *x*_*i *_denotes the genotype (coded as 0, 1, 2, corresponding to *AA*, *AB*, and *BB*) of the *i*^th ^observation. We consider two measures of explained variation for binary outcomes [7]. These measures are based on direct and indirect estimates of predictive accuracy. The direct measure is based on residual from the fit and the indirect index is related to a standard measure of information. Let the estimates from a logistic model without a covariate be  and the estimates from a model with covariate *x*_*i *_be . Define  and , and the explained variation based on the direct estimates becomes . Similarly, let  and , then the explained variation based on indirect estimates is calculated as . We denote these two approaches as DirEV and IndirEV, respectively. In the models which include principal components to adjust for population stratification, the explained variation attributable to each SNP were obtained.

### Pathway-based analysis

We summarize the steps for our method as follows (adapted from Wang et al. [8]):

#### 1. Obtain test/summary statistic

For each SNP, we computed one of the following: measure of explained variation and univariate SNP association test (*χ*^2^) where population stratification is adjusted for by including 15 PCs.

#### 2. Map SNPs to genes

We obtained the nearest gene name for each SNP from the Illumina SNP annotation file (HumanHap650Yv3_Gene_Annotation.txt, available from https://icom.illumina.com/) based on physical distance. The 490,000 SNPs were mapped to 16,500 genes.

#### 3. Aggregate test/summary statistic

For each gene, we obtained an aggregate summary measure or test statistic based on individual values (from Step 1 above) for SNPs assigned to this gene (Step 2). Here we used the *maximum summary measure or maximum test statistic *over all SNPs mapped to the gene.

4. The aggregated summary measure or test statistics were used to evaluate the significance of predefined gene sets/pathways [9] based on the gene set enrichment analysis (GSEA) method [10]. Here we used the c2 curated gene sets, which are obtained from online pathway databases, citation in PubMed [9], and knowledge of domain experts and included 1900 gene sets collected from canonical pathways, chemical and genetic perturbations, BioCarta pathways, GeneMAPP [11], and KEGG [12]. A Kolmogorov-Smirnov non-parametric rank statistic was performed using the GseaPreranked tool included in the GSEA software. Gene sets were ranked by their FDR *q*-value, where the *q*-value of a test measures the proportion of false positives incurred (FDR) when that particular test is called significant. The empirical null distribution was obtained using 1000 random permutations. We defined a given gene set as significantly enriched in the data if it has FDR *q*-value of less than 0.05.

## Results

For the 1900 gene sets that we evaluated, all three methods (Chi-Sq, DirEV and IndirEV) identified 10 gene sets that have FDR *q*-value of less than 0.05. Table 1 shows these ten significantly enriched gene sets/pathways identified by GSEA for each of the three methods. As seen from Table 1, the ten significantly enriched pathways for the three summary methods are the same. The pathways identified using DirEv and IndirEV have similar FDR *q*-value and ranks. The FDR *q*-value results based on the *χ*^2 ^tests are slightly different from those based on DirEV and IndirEV.

**Table 1:** Significant pathways identified by three test statistic methods

		*χ* ^2^		DirEV		IndirEV	
				
Gene sets/pathways	No. genes^a^	Rank^b^	FDR *q*-value	Rank	FDR *q*-value	Rank	FDR *q*-value
Hsa04612 antigen processing and presentation	57	1	<1*10^-8^	1	<1*10^-8^	1	<1*10^-8^
Hsa04940 Type I diabetes mellitus	40	2	<1*10^-8^	2	<1*10^-8^	2	<1*10^-8^
Wieland hepatitis B-induced	87	3	<1*10^-8^	3	0.004	3	0.001
Ctla4 pathway	18	4	6*10^-5^	6	0.006	4	0.003
Ami pathway	22	5	0.01	7	0.006	10	0.015
Csk pathway	22	6	0.01	10	0.011	7	0.012
Sana Ifng endothelial up	67	7	0.011	8	0.006	9	0.013
Th1th2 pathway	17	8	0.011	9	0.009	8	0.013
Inflam pathway	28	9	0.012	4	0.005	6	0.006
Hsa04514 cell adhesion molecule	115	10	0.02	5	0.005	5	0.004

^a^Number of genes found in our data. (The actual number of genes defined in the set is larger than these numbers.)

^b^The rank is based on the FDR *q*-value for all 1,900 tested gene sets in each method.

As discussed before, there are few potential genes associated with RA: *PTPN22 *on chromosome 1, *HLA_DRB1 *on the MHC region on chromosome 6, and *TRAF1/C5 *on chromosome 9. However, *PTPN22 *and *TRAF1/C5 *were not in any of the ten significantly enriched gene sets. Therefore, we further evaluated the distributions of genes and SNPs in the MHC region compared with the rest of the genome (those not in MHC region) in these ten gene sets/pathways (Table 2). To do this, we defined the MHC region as 29-34 Mb on chromosome 6. The region is defined based on the results shown in Supplementary Table 1B of Plenge et al. [3], that is, SNPs that have *p*-value < 0.0001. We found 561 SNPs in the defined MHC region with *p*-value < 0.0001; and 227 genes in the region using BioMart [13].

Table 2 shows the distribution of these genes and SNPs in the defined MHC region and other genes and SNPs with *p*-value < 0.0001 in the rest of the genome in the 10 gene sets/pathways. It can be seen the MHC region is enriched for genes from the following three gene sets: "Hsa04612 Antigen Processing and Presentation," "Hsa04940 Type I Diabetes Mellitus," and "Hsa04514 Cell Adhesion Molecule." There were 22 HLA-related genes in common among these three gene sets. Of these, 20 were found in the defined MHC region. Five SNPs with *p*-value < 0.0001 were present in these three gene sets in the MHC region. Interestingly, one SNP (rs1018361) with *p*-value of 2.6 × 10^-5 ^on chromosome 2 was present in one of the three gene sets ("Hsa04514 Cell Adhesion Molecule") in the rest of genome (the region excluding MHC region) while the other two gene sets had no significant SNPs in the rest of the genome.

**Table 2:** Distribution of selected genes and SNPs in each of the two regions and nine gene sets/pathways

	MHC region (Chr 6, 29-34 Mb^a^)			Rest of genome		
		
Gene sets/pathways	No. genes	%^b^	#SNPs	No. genes	%^a^	No. SNPs
Hsa04612 antigen processing and Presentation	24	42.1	5	33	57.9	0
Hsa04940 Type I Diabetes Mellitus	22	55.0	6	18	45.0	0
Wieland hepatitis B-iInduced	15	17.2	4	72	82.8	0
Ctla4 pathway	2	11.1	0	16	88.9	1
Ami pathway	2	9.1	0	20	90.9	0
Csk pathway	2	9.1	0	20	90.9	0
Sana Ifng endothelial up	11	16.4	2	56	83.6	0
Th1th2 pathway	2	11.8	0	15	88.2	0
Inflam pathway	4	14.3	2	24	85.7	0
Hsa04514 cell adhesion molecule	20	17.4	5	95	82.6	1

^a^This region covers approximately 0.2% the whole genome

^b^The percentage of genes in a given gene set that were found in the region

The significant SNP on chromosome 2 (rs1018361) may be of interest for further investigation because the closest gene (*CTLA4*) to this SNP shares the same pathway as those genes containing the MHC region. Moreover, previous association study showed that RA is associated with *CTLA4 *[2]. Similarly, the cell adhesion molecules (proteins) may have an important role in regulation of the RA development than other tested pathways. As shown in Table 2, both genes and SNPs with *p*-value < 0.0001 in the pathway (cell adhesion molecule) are found in both the MHC region and the rest of the genome, while other significant pathways have SNPs with *p*-value < 0.0001 only in the MHC region or are not found in either the MHC region or the rest of the genome.

## Conclusion

Overall, using a pathway-based analysis, we found some of the significant gene sets/pathways were enriched in the well known MHC region associated with RA. However, some of the loci that have been reported to be related to RA, such as *PTPN22 *and *TRAF1/C5*, were not found to be enriched in any of the gene sets/pathways we identified as significant. Our results also showed that pathway-based analysis may identify other RA-related loci (e.g., rs1018361) because the gene (*CTLA4*) closest to this SNP has previously been shown to be associated with RA and the significant pathways identified here contained this gene.

## List of abbreviations used

DirEV: Explained variation based on the direct estimates; FDR: False-discovery rate; GSEA: Gene set enrichment analysis; IndirEV: Explained variation based on indirect estimates; MHC: Major histocompatability complex; PC: Principal coordinates; RA: Rheumatoid arthritis; SNP: Single-nucleotide polymorphism.

## Competing interests

The authors declare that they have no competing interests.

## Authors' contributions

JB initiated the study and proposed the pathway-based analysis ideas and methods for genetic association analysis. PH performed all of the data analysis and drafted the paper with JB, JSH, and ADP. JSH, ADP, EP, and DT contributed by providing critical comments which helped with refining the methods, analyses, and interpretation of the data. All authors read and approved the final manuscript.
